# Re: Evaluation of a telemedicine-based training for final-year medical students including simulated patient consultations, documentation, and case presentation

**DOI:** 10.3205/zma001509

**Published:** 2021-11-15

**Authors:** Sigrid Harendza

**Affiliations:** 1 Universitätsklinikum Hamburg-Eppendorf, III. Medizinische Klinik, Hamburg, Germany

**Keywords:** medical education, COVID-19, coronavirus, pandemic, online teaching

## Authors’ reply

Dear Mister Majeed,

thank you for your interest in our project and your appreciation that we provide our training of medical competences to final-year medical students in a telemedicine format since the beginning of the COVID-19 pandemic [1]. We were very happy that the students’ learning experiences were equally good compared to a similar training in a presence format [2]. As you rightly point out, it is very important for evaluations that Likert scales are unambiguously worded to provide solid results. Sometimes, ambiguity can occur, for example, when Likert scales are translated [3]. In the translation of our Likert scale from German to English, “fully applies” means, I “strongly agree” to the evaluation item. However, we agree that the phrase “agree” is less ambiguous than “apply” and we actually used agreement for the Likert scales of our other instruments, e.g. the ComCare scale for measuring medical communication and interpersonal skills [4]. It measures “language”, “listening”, “interest”, “needs”, “compassion”, “next steps”, and “atmosphere” [5]. Whether, and if so, what differences occurred between the presence and telemedicine formats is currently a goal of our further research. Therefore, we cannot assess yet whether a possible difference in the nonverbal communication of the participating students or an awkwardness in repeating sensitive information occurred.

Studying a pre-recorded “real” consultation as reference is certainly as powerful a learning tool as the so called “sitting in” as an observer in a presence situation [6], and as recording and playback of a clinical situation, like you describe from the “Clinical Communication Course” at the School of Clinical Medicine in Cambridge. Since we discovered that simulated patients’ ComCare scale ratings differed from the internal and external patient perspective [7] we are planning further learning opportunities for the training participants where they can watch their own consultation videos and receive personal feedback from the simulated patients and attendings. Thank you again for the suggestions on medical teaching. We hope to develop further appropriate formats for remote learning with our training.

Yours sincerely,

Sigrid Harendza (on behalf of all authors)

## Competing interests

The author declares that she has no competing interests.

## References

[1] Harendza S, Gärtner J, Zelesniack E, Prediger S (2020). Evaluation of a telemedicine-based training for final-year medical students including simulated patient consultations, documentation and case presentation. GMS J Med Educ.

[2] Prediger S, Schick K, Fincke F, Fürstenberg S, Oubaid V, Kadmon M, Berberat PO, Harendza S (2020). Validation of a competence-based assessment of medical students' performance in the physician's role. BMC Med Educ.

[3] Granas AG, Nørgaard LS, Sporrong SK (2014). Lost in translation?: Comparing three Scandinavian translations of the beliefs about medicines questionnaire. Patient Educ Couns.

[4] Gärtner J, Bußenius L, Schick K, Prediger S, Kadmon M, Berberat PO, Harendza S (2021). Validation of the ComCare index for rater-based assessment of medical communication and interpersonal skills. Patient Educ Couns.

[5] Gärtner J, Prediger S, Harendza S (2021). Development and pilot test of ComCare - a questionnaire for quick assessment of communicative and social competences in medical students after interviews with simulated patients. GMS J Med Educ.

[6] Spencer J (2003). ABC of teaching and learning in medicine: Learning and teaching in the clinical environment. BMJ.

[7] Prediger S, Harendza S (2021). Perspective matters: assessment of medical students' communication and interpersonal skills by simulated patients from the internal and external patient perspective. GMS J Med Educ.

